# Supplementary material for: PD-L2 controls peripherally induced regulatory T cells by maintaining metabolic activity and Foxp3 stability
Source: Nat Commun. 2022 Aug 31;13:5118. doi: 10.1038/s41467-022-32899-5 (PMC9433378; doi:10.1038/s41467-022-32899-5)
Supplement: Supplementary file 1 — Supplementary Information [file 41467_2022_32899_MOESM1_ESM.pdf]

PD-L2 controls peripherally induced regulatory T cells by maintaining metabolic activity and Foxp3 stability

Benjamin P. Hurrell[1], Doumet Georges Helou[1], Emily Howard[1], Jacob Painter[1], Pedram Shafiei-Jahani[1], Arlene H. Sharpe[2] and Omid Akbari[1*]

Supplementary Material

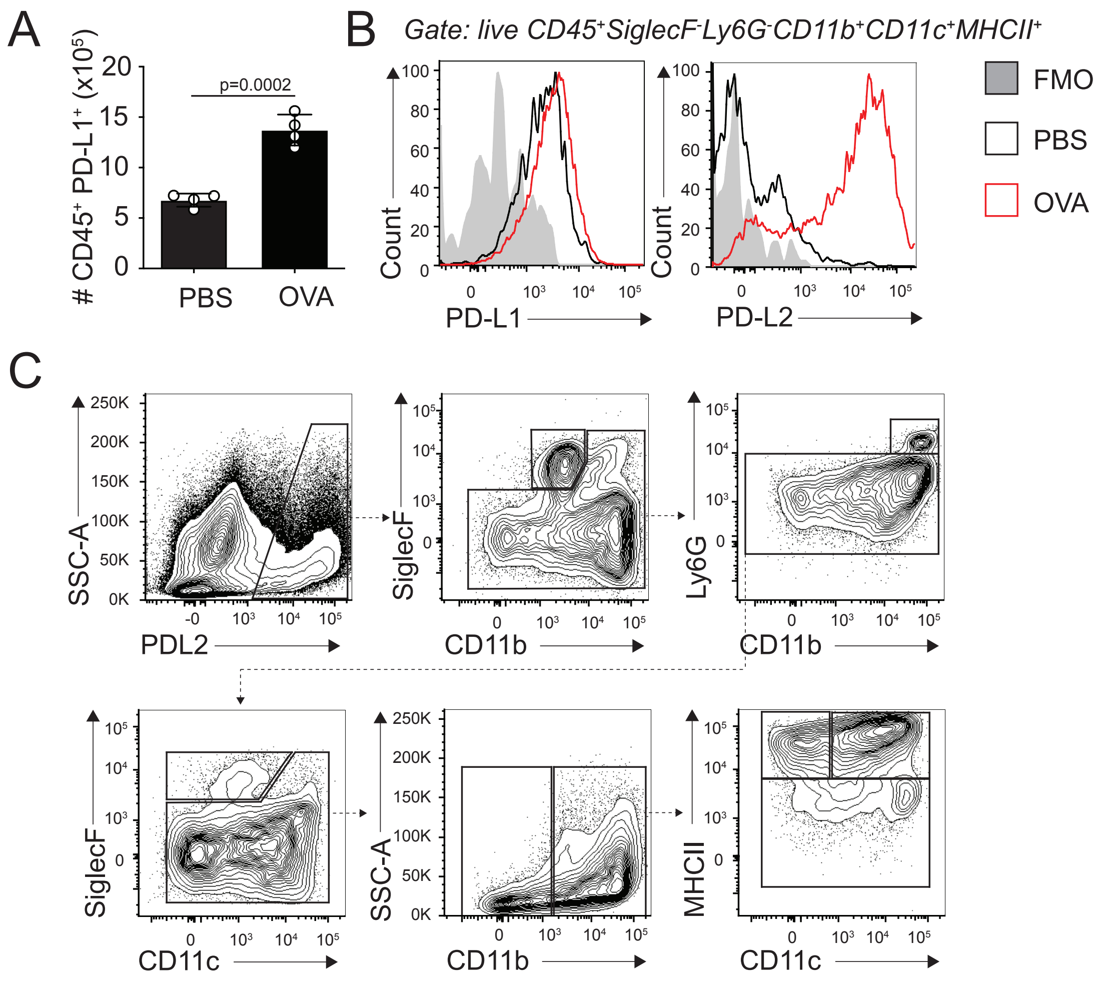

Supplementary Figure 1 – PD-L1 and PD-L2 expression in the lungs.

(A) BALB/c (WT) mice were intranasally challenged on 3 consecutive days with 100µg ovalbumin (OVA) or PBS. On day 4, lungs were collected and the expression of PD-L1 measured by flow cytometry. Number of CD45$^+$ PD-L1$^+$ lung cells presented in numbers of cells +/- SEM.

(B) Representative flow cytometry plots of PD-L1 and PD-L2 expression within CD45$^+$SiglecF$^+$Ly6G$^+$CD11b$^+$CD11c$^+$MHCII$^+$ cells. FMO: “full minus one” staining control.

(C) Gating strategy used for myeloid cells in the lungs.

Data are representative of at least 2 independent experiments and are presented as means ± SEM, n=4 biologically independent mice. Source data are provided as a Source Data file. A two-tailed Student’s t test for unpaired data was applied for comparisons between two groups.

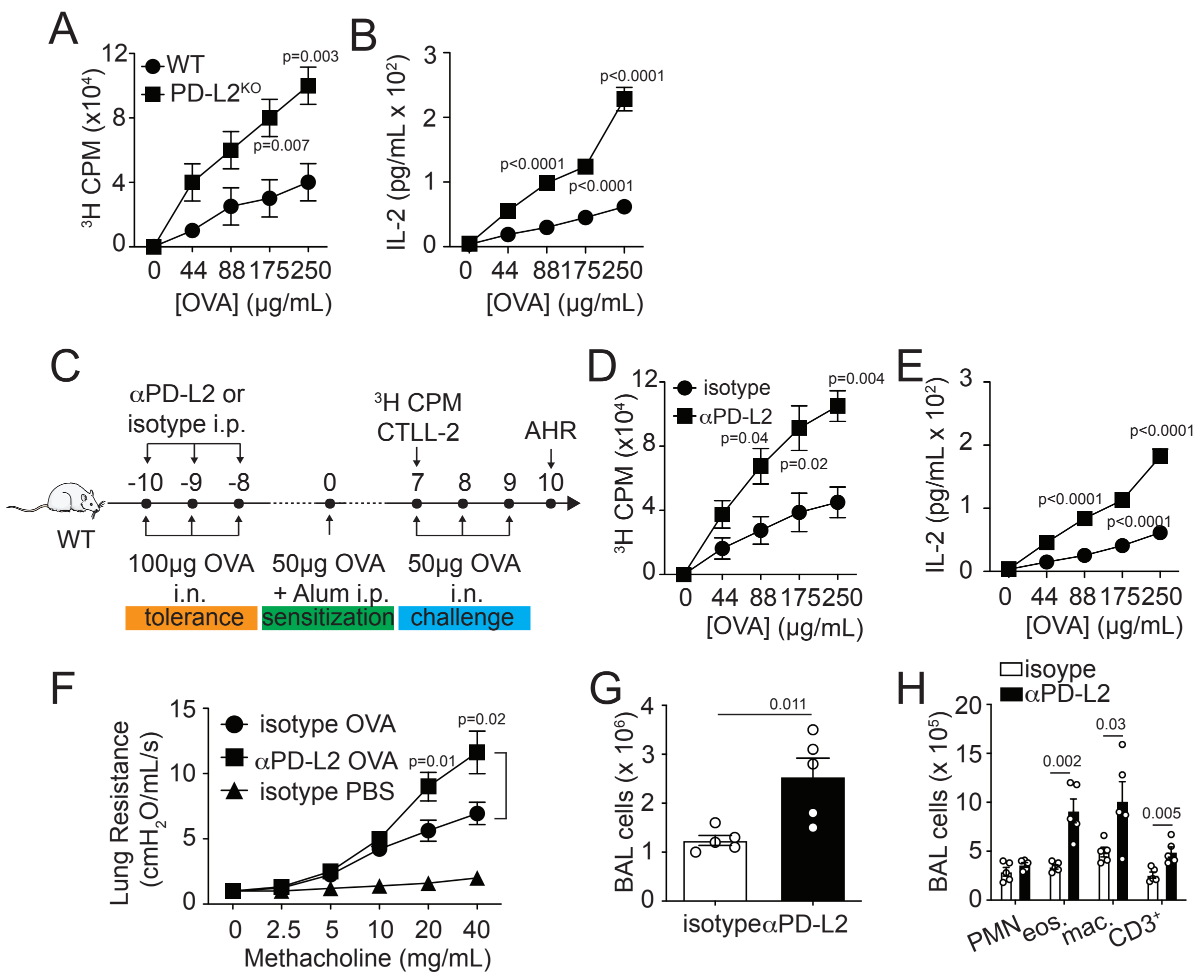

Supplementary Figure 2 – PD-L2 blockade inhibits the induction of respiratory tolerance.

(A-B) BALB/c (WT) and PD-L2^{KO} mice were i.n. challenged on days -10, -9 and -8 with 100µg ovalbumin (OVA). On day 0, mice were intraperitoneally sensitized with 50µg OVA emulsified in alum (1:1).

(A) Ci ^{3}H thymidine incorporation in splenocytes on day 7 in response to increasing doses of OVA-peptide. n=5 biologically independent samples.

(B) Levels of IL-2 in the splenocyte culture supernatants on day 7 in response to increasing doses of OVA-peptide. n=5 biologically independent samples.

(C) BALB/c (WT) mice were i.n. challenged on days -10, -9 and -8 with 100µg ovalbumin (OVA) and treated with 250µg intraperitoneally with aPD-L2 (3.2) antibody or isotype control. On day 0, mice were intraperitoneally sensitized with 50µg OVA emulsified in alum (1:1) and on days 7, 8 and 9 mice were further i.n. challenged with 50µg OVA i.n.

(D) Ci ^3H thymidine incorporation in splenocytes at day 7 in response to increasing doses of OVA-peptide. n=4 biologically independent samples.

(E) Levels of IL-2 in the splenocyte culture supernatants at day 7 in response to increasing doses of OVA-peptide. n=5 biologically independent samples.

(F) Lung resistance in response to increasing doses of methacholine measured in restrained ventilated mice at day 10.

(G) Number of total $CD45^+$ bronchoalveolar lavage (BAL) fluid cells.

(H) Numbers of $CD11b^+$ $Ly6G^+$ neutrophils (PMN), $CD11c^-$ $SiglecF^+$ eosinophils (eos.), $CD11c^+$ $SiglecF^+$ $CD64^+$ macrophages (mac.) and $CD3^+$ T-cells. n=5 biologically independent samples.

Data are representative of 2 independent experiments and are presented as means ± SEM. Source data are provided as a Source Data file. A two-tailed Student's t test for unpaired data was applied for comparisons between two groups, except for multi-group comparisons where Tukey's multiple comparison one-way ANOVA tests were used. Mouse image provided with permission from Servier Medical Art.

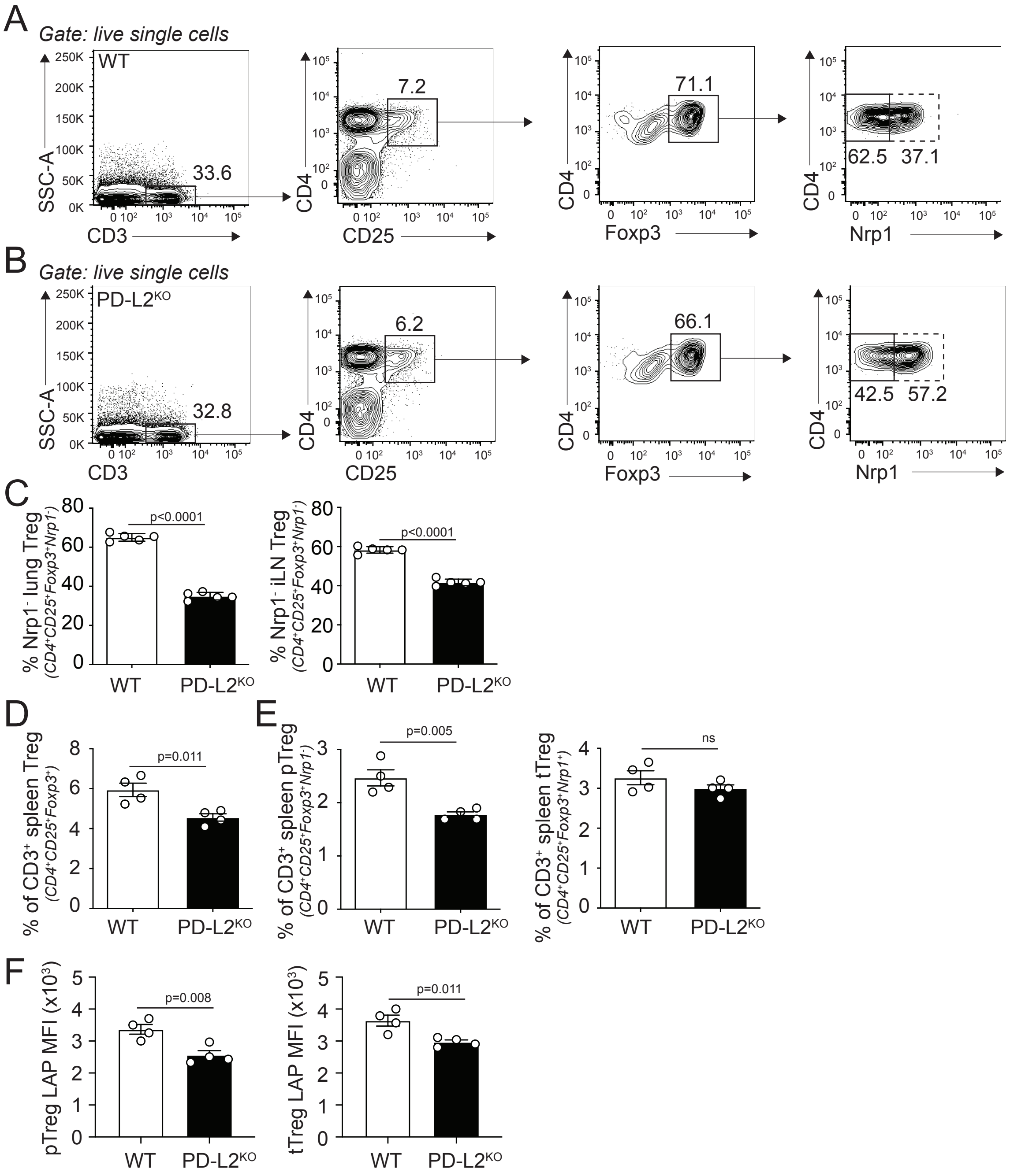

Supplementary Figure 3 – spleen Tregs under homeostatic conditions

Spleens of Foxp3^{GFP} and PD-L2^{KO} Foxp3^{GFP} mice were collected and analyzed by flow cytometry. **(A)** Representative flow cytometry plots of CD3^{+}CD4^{+}CD25^{+}Foxp3^{GFP+}Nrp1^{-} pTregs and CD3^{+}CD4^{+}CD25^{+}Foxp3^{+}Nrp1^{+} tTregs within Foxp3^{GFP} WT mice.

(B) (A) Representative flow cytometry plots of $CD3^{+}CD4^{+}CD25^{+}Foxp3^{GFP+}Nrp1^{-}$ pTregs and $CD3^{+}CD4^{+}CD25^{+}Foxp3^{+}Nrp1^{+}$ tTregs within PD-$L2^{KO}$ $Foxp3^{GFP}$ Tregs.

(C) Frequencies of $CD3^{+}CD4^{+}CD25^{+}Foxp3^{GFP+}Nrp1^{-}$ pTregs in lungs and inguinal lymph nodes (iLN) of $Foxp3^{GFP}$ and PD-$L2^{KO}$ $Foxp3^{GFP}$ mice, n=5 biologically independent mice.

(D) Frequencies within $CD3^{+}$ cells of total $CD3^{+}CD4^{+}CD25^{+}Foxp3^{GFP+}$ Tregs, n=4 biologically independent mice.

(E) Frequencies within $CD3^{+}$ cells of $CD3^{+}CD4^{+}CD25^{+}Foxp3^{GFP+}Nrp1^{-}$ pTregs and $CD3^{+}CD4^{+}CD25^{+}Foxp3^{GFP+}Nrp1^{+}$ tTregs, n=4 biologically independent mice.

(F) $CD3^{+}CD4^{+}CD25^{+}Foxp3^{GFP+}Nrp1^{-}$ pTregs and $CD3^{+}CD4^{+}CD25^{+}Foxp3^{GFP+}Nrp1^{+}$ tTregs were FACS-sorted from the spleens of $Foxp3^{GFP}$ and PD-$L2^{KO}$ $Foxp3^{GFP}$ mice and cultured for 24 hours with CD3/CD28 beads, IL-2 and TGF-β. LAP MFI was measured by flow cytometry, n=4 biologically independent samples. Data are representative of 2 independent experiments and presented as mean +/- SEM. Source data are provided as a Source Data file. A two-tailed Student's t test for unpaired data was applied for comparisons between two groups.

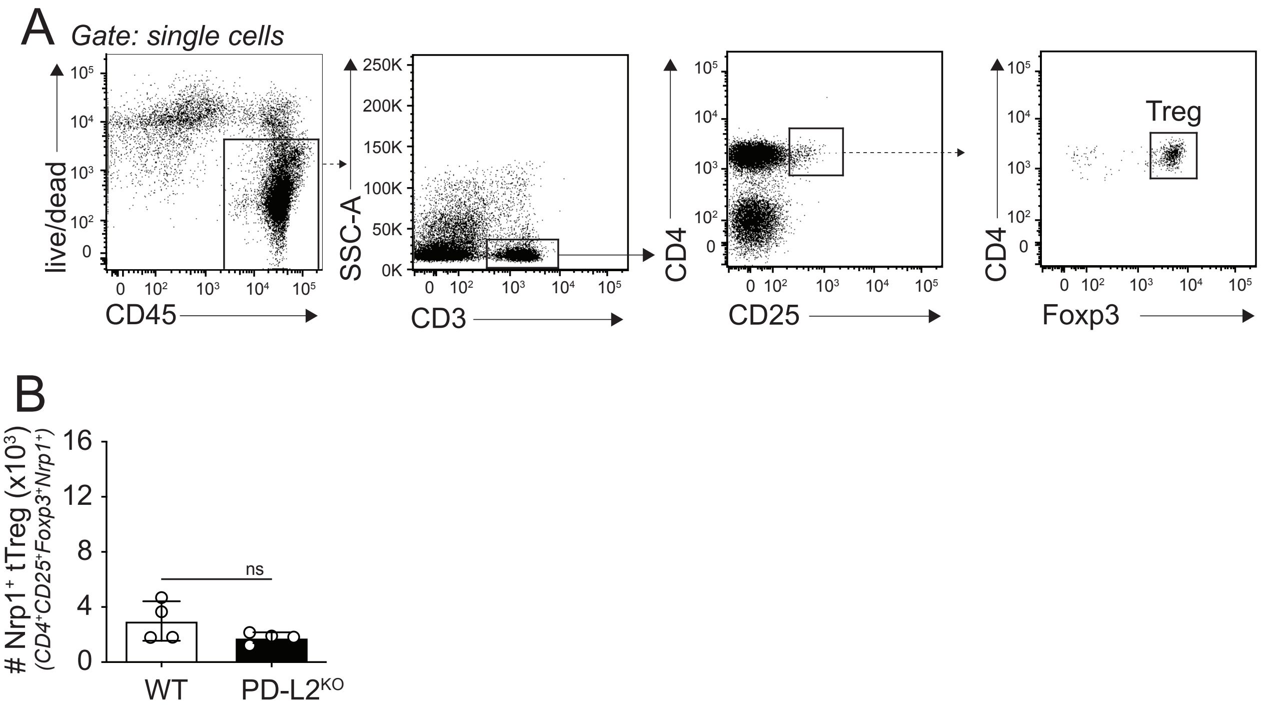

Supplementary Figure 4 – Lung Treg gating strategy and nTreg numbers

Foxp3^{GFP} and PD-L2^{KO} Foxp3^{GFP} mice were i.n. challenged on days -10, -9 and -8 with 100µg ovalbumin (OVA). On day 0, mice were intraperitoneally sensitized with 50µg OVA emulsified in alum (1:1) and iTreg numbers were analyzed by flow cytometry on day 7.

(A) Lung Treg gating strategy.

(B) Absolute numbers of Nrp1^{+} nTregs, n=4 biologically independent mice. Data are representative of 2 independent experiments and presented as mean +/- SEM. Source data are provided as a Source Data file. A two-tailed Student's t test for unpaired data was applied for comparisons between two groups.

**Antibody and clone**	**Source**	**cat#**	**dilution**
PECy7 anti-mouse **CD45** (30-F11)	BioLegend	103114	1-300
APCCy7 anti-mouse CD45 (30-F11)	BioLegend	103116	1-300
PerCPCy5.5 anti-mouse **CD3** (17A2)	BioLegend	100218	1-300
FITC anti-mouse **CD3** (17A2)	BioLegend	100204	1-300
BV421 anti-mouse **CD4** (GK1.5)	BioLegend	100438	1-300
APCCy7 anti-mouse **CD4** (GK1.5)	BioLegend	100414	1-300
PECy7 anti-mouse **CD8a** (53-6.7)	BioLegend	100722	1-300
APCCy7 anti-mouse **CD25** (PC61)	BioLegend	102026	1-300
BV421 anti-mouse **CD25** (PC61)	BioLegend	102034	1-300
BV510 anti-mouse **CD25** (PC61)	BioLegend	102042	1-300
PE anti mouse **CD304** (Neuropilin-1, 3E12)	BioLegend	145204	1-300
APC anti-mouse **CD62L** (MEL-14)	BioLegend	104412	1-300
APCCy7 anti-mouse **CD44** (IM7)	BioLegend	103028	1-300
PECy7 anti-mouse **CD44** (IM7)	BioLegend	103030	1-300
PerCPCy5.5 anti-mouse **TCR DO11.10** (KJ1-26)	BioLegend	118512	1-300
APCCy7 anti-mouse **CD11c** (N418)	BioLegend	117324	1-300
PerCPCy5.5 anti-mouse **CD11c** (N418)	BioLegend	117328	1-300
APC anti-mouse **CD170** (SiglecF, S17007L)	BioLegend	155507	1-300
PECy7 anti-mouse **Ly6G** (1A8)	BioLegend	127618	1-300
BV510 anti mouse **I-A/I-E** (M5/114.15.2)	BioLegend	107636	1-300
APC anti-mouse **CD274** (PD-L1, 10F9G2)	BioLegend	124312	1-300
PECy7 anti-mouse **CD273** (PD-L2, TY25)	BioLegend	107214	1-300
BV421 anti-mouse **CD279** (PD-1, 29F1A12)	BioLegend	135218	1-300
FITC anti-mouse **CD19** (6D5)	BioLegend	115506	1-300
APC anti-mouse Gr-1 (RB6-8C5)	BioLegend	108412	1-300
FITC anti-mouse **Foxp3** (MF-14)	BioLegend	126406	1-100
PE anti-mouse **CD170** (SiglecF, E50-2440)	BD Biosciences	552126	1-300
eFluor450 anti-mouse **CD11b** (M1/70)	Thermofisher	48-0112-82	1-300

Supplementary Table 1 - Antibody catalog numbers and dilutions
